# Supplementary material for: The effectiveness of savouring interventions on well-being in adult clinical populations: A protocol for a systematic review
Source: PLoS One. 2024 Apr 16;19(4):e0302014. doi: 10.1371/journal.pone.0302014 (PMC11020756; doi:10.1371/journal.pone.0302014)
Supplement: S1 File — (DOCX) [file pone.0302014.s002.docx]

S2. Supplementary file. Search strategies for electronic databases.

**Search Strategies for all Databases**

***Search strategy: PsycINFO***

*#1 AB (savoring OR savouring OR “positive reminisc*” OR basking OR “positive life review” OR “behavioural expression” OR “memory building” OR “self-congratulation” OR “positive anticipation” OR “positive emotion regulation” OR “positive anticipation” OR appreciation OR “mindful attention” OR awe OR wonder OR luxuriat* OR bask* OR thanksgiving OR marvel* OR “counting blessings” OR “three good things”)*

*#2 AB (DE “positive reminiscence” OR DE “life review”)*

*#3 #1 OR #2*

*#4 AB (intervention* OR therap* OR treatment* OR program*)*

*#5 AB (DE “Intervention” OR DE “Therapy” OR DE “Psychotherapy”)*

*#6 #4 OR #5*

*#7 AB (“randomised controlled trial” OR “randomised control trial” OR “randomized controlled trial” OR “randomized control trial” OR “RCT”)*

*#8 AB #3 AND #6 AND #7*

***Search strategy: PubMed***

*#1 (savoring [tiab] OR savouring [tiab] OR “positive reminisc*” [tiab] OR basking [tiab] OR “ positive life review” OR “behavioural expression” [tiab] OR “memory building” [tiab] OR “self-congratulation” [tiab] OR “positive anticipation” [tiab] OR “positive emotion regulation” [tiab] OR “positive anticipation” [tiab] OR appreciation [tiab] OR “mindful attention” [tiab] OR awe [tiab] OR wonder [tiab] OR luxuriat* [tiab] OR bask* [tiab] OR thanksgiving [tiab] OR marvel* [tiab] OR “counting blessings” [tiab] OR “three good things” [tiab])*

*#2 (emotional regulation [mh])*

### *#3 #1 OR #2*

*#4 (intervention*[tiab] OR therap*[tiab] OR treatment*[tiab] OR program*[tiab])*

*#5 therapy [mh]*

*#6 #4 OR #5*

*#7 (“randomised controlled trial” [tiab] OR “randomised control trial” [tiab] OR “randomized controlled trial” [tiab] OR “randomized control trial” [tiab] OR “RCT” [tiab])*

*#8 #3 AND #6 AND #7*

***Search strategy: CINAHL***

*#1 AB (savoring OR savouring OR “positive reminisc*” OR basking OR “positive life review” OR “behavioural expression” OR “memory building” OR “self-congratulation” OR “positive anticipation” OR “positive emotion regulation” OR “positive anticipation” OR appreciation OR “mindful attention” OR awe OR wonder OR luxuriat* OR bask* OR thanksgiving OR marvel* OR “counting blessings” OR “three good things”)*

*#2 AB (DE “life history review” OR DE “reminiscence therapy”)*

*#3 #1 OR #2*

*#4 AB (intervention* OR therap* OR treatment* OR program*)*

*#5 AB (“randomised controlled trial” OR “randomised control trial” OR “randomized controlled trial” OR “randomized control trial” OR “RCT”*

*#8 AB #3 AND #4 AND #5*

***Search strategy: Scopus***

*#1 TITLE-ABS-KEY({savoring} OR {savouring} OR {positive reminisc*} OR {basking} OR {positive life review} OR {behavioural expression} OR {memory building} OR {self-congratulation} OR {positive anticipation} OR {positive emotion regulation} OR {positive anticipation} OR {appreciation} OR {mindful attention} OR {awe} OR {wonder} OR {luxuriat*} OR {bask*} OR {thanksgiving} OR {marvel*} OR {counting blessings} OR {three good things})*

*#2 TITLE-ABS-KEY (intervention* OR therap* OR treatment* OR program*)*

*#3 TITLE-ABS-KEY ({randomised controlled trial} OR {randomised control trial} OR {randomized controlled trial} OR {randomized control trial} OR {RCT})*

*#4 #1 AND #2 AND #3*

*Limit to subject areas of Medicine, Psychology & Social Sciences*

*Limit by document type “Article”*

First 200 hits from Google Scholar after inputting to search bar:

*(savoring OR savouring OR “positive reminisc*” OR basking OR “positive life review” OR “behavioural expression” OR “memory building” OR “self-congratulation” OR “positive anticipation” OR “positive emotion regulation” OR “positive anticipation” OR appreciation OR “mindful attention” OR awe OR wonder OR luxuriat* OR bask* OR thanksgiving OR marvel* OR “counting blessings” OR “three good things”)*
