# Supplementary material for: The effectiveness of savouring interventions on well-being in adult clinical populations: A protocol for a systematic review
Source: PLoS One. 2024 Apr 16;19(4):e0302014. doi: 10.1371/journal.pone.0302014 (PMC11020756; doi:10.1371/journal.pone.0302014)
Supplement: S2 File — (DOCX) [file pone.0302014.s003.docx]

S3. Supplementary file. Screening tool for paper eligibility.

**Screening Tool**

All criteria must be met for a paper to be eligible. If any criteria is absent, the paper is to be excluded:

| **Inclusion Criteria** | **Present** | **Absent** |
| --- | --- | --- |
| **Design:**  Randomised Controlled Trial |  |  |
| **Participants:**  Age (≥ 18 years)  Clinical population (mental or physical health diagnosis given by medical professionals and/or based on meeting significant cut off points on validated psychometric instruments) |  |  |
| **Intervention:**  Savouring interventions. These include:   - Interventions which instruct participants to attempt to attend to, intensify and prolong positive emotions or positive affect associated with a positive stimulus, whether that be real or imagined. - May emphasise the development of savouring future positive events before they occur (anticipation), present positive events while they are unfolding (savouring the present moment), or past positive events after they occur (reminiscence). - Must instruct participants to attend mindfully, reminisce, review, anticipate positive events, experiences or memories only (not integrate meaning from negative or neutral events). - Examples include: thinking, talking or writing about positive events, counting blessings, noticing ‘Three Good Things’, reflecting on kindness (done to and performed by the individual) or engaging in any specific strategy to increase positive emotions attached to an event or the present moment e.g. implementing temporal scarcity into everyday experiences or enhancing active-constructive communications with others. |  |  |
| **Comparisons:**  Waiting list control group or alternative intervention control group (e.g. treatment as usual, placebo control groups, or another well-defined treatment such as cognitive behavioural therapy) |  |  |
| **Outcome measures:**  Validated measures of well-being (happiness, life satisfaction), quality of life, depression, anxiety or stress |  |  |
